# Biological and transcriptomic studies reveal *hfq* is required for swimming, biofilm formation and stress response in *Xanthomonas axonpodis* pv. *citri*

**DOI:** 10.1186/s12866-019-1476-9

**Published:** 2019-05-22

**Authors:** Xuelu Liu, Yuping Yan, Haodi Wu, Changyong Zhou, Xuefeng Wang

**Affiliations:** 1 National Engineering Research Center for Citrus, Citrus Research Institute, Southwest University/Chinese Academy of Agricultural Sciences, Chongqing, 400712 People’s Republic of China; 2 Present address: Agriculture commission of Guangan district, Guangan, Sichuan China

**Keywords:** *Xanthomonas axonpodis* pv. *citri*, *Xanthomonas citri* subsp. *citri*, *hfq* gene, Biofilm

## Abstract

**Background:**

Hfq is a widely conserved bacterial RNA-binding protein which generally mediates the global regulatory activities involv ed in physiological process and virulence. The goal of this study was to characterize the biological function of *hfq* gene in *Xanthomonas axonpodis* pv. *citri* (*Xac*), the causal agent of citrus canker disease.

**Results:**

An *hfq* mutant in *Xac* was generated by plasmid integration. The loss of *hfq* resulted in attenuation of bacterial growth, motility and biofilm formation. In addition, the *hfq* mutation impaired *Xac* resistance to H_2_O_2_ and both high and low pH environments, but did not affect the virulence to citrus. RNA-Seq analyses indicated that Hfq played roles in regulating the expression of 746 genes. In *hfq* mutant, gene expression related to chemotaxis, secretion system, two-component system, quorum sensing and flagellar assembly were repressed, whereas expression of ribosomal genes were significantly up-regulated. The down-regulated expression of three bacterial chemotaxis related genes and seven flagella genes, which involved in cell growth and biofilm formation, were further validated by RT-qPCR.

**Conclusions:**

The study demonstrated that *hfq* was involved in multiple biological processes in *Xac*. The results could serve as initiate points for identifying regulatory sRNAs and genes controlled by Hfq-sRNA interactions in *Xac*.

**Electronic supplementary material:**

The online version of this article (10.1186/s12866-019-1476-9) contains supplementary material, which is available to authorized users.

## Background

The RNA chaperone Hfq was originally discovered in *Escherichia coli* as a host factor essential for replication of the bacteriophage Qβ [1, 2] and afterwards identified in a large number of Gram-positive and Gram-negative bacterial species [3]. Hfq forms a hexameric ringshaped doughnut structure that mediates the global post-transcriptional regulation involved in numerous physiological and biochemical functions in bacteria [2]. Inactivation of *hfq* gene exhibits broadly pleiotropic phenotypes in *Escherichia coli*, e.g. alteration in growth rate and tolerance to UV or high osmolarity stresses [4]. Many *hfq* mutants from a broad spectrum of bacterial pathogens show a general role in bacterial physiology and virulence [3, 5–7]. Notably, *hfq* deficiency impairs the stability and functional activation of many Hfq-dependent small non-coding RNAs (sRNAs) which are usually encoded in the intergenic regions of bacterial genomes [8]. The majority of these Hfq-dependent sRNAs can modulate the expression of target mRNAs by base pairing mechanisms, and then affect the downwards cellular processes [9, 10]. Thus, Hfq protein in facilitating the interaction between small non-coding RNAs (sRNAs) and target mRNAs is currently considered as its most prominent function.

*Xanthomonas* spp. are economically important phytopathogens and are grouped into pathovars (pv.) based on their specific host ranges [11, 12]. *X. oryzae* pv. *oryzae* (*Xoo*), *X. campestris* pv. *vesicatoria* (*Xcv*), *X. campestris* pv. *campestris* (*Xcc*) and *X. axonpodis* pv. *citri* (*Xac*) (synonym *X. citri* subsp. *citri*) are economical important pathogens among the genus *Xanthomonas*. *hfq* is conserved in *Xanthomonas* spp. and has been investigated in *Xoo* and *Xcv*. Inactivation of *hfq* in *Xoo* affects it growth in complex medium, but does not disrupt its virulence [13]. Similarly, *hfq* gene in *Xcv* does not involve in virulence [14]. Although many Hfq-dependent sRNAs were identified in *Xcv*, *Xcc*, and *Xoo* [15–17], their involvements in regulation and virulence function have been poorly characterized. *Xanthomonas axonpodis* pv. *citri* (*Xac*), the causal agent of citrus canker, is an important bacterial pathogen that severely affects citrus production worldwide. The two key regulators, HrpG and HrpX, regulating type III secretion system (T3SS), are known to play a critical role in *Xac* infection [16]. However, the roles of global regulator *hfq* and its related sRNAs remain to be determined.

In this study, the function of *hfq* in *Xac* biology and gene expression was characterized by using an *hfq* mutant constructed in strain *Xac*29–1. The inactivation of *hfq* resulted in the phenotypic alterations in bacterial growth, swimming motility, biofilm formation and stress response. Results of RNA-Seq analyses indicated that Hfq plays an important role in multiple biological processes including chemotaxis, flagellar assembly and secretion systems.

## Results

### The deletion of *hfq* attenuates the *Xac* growth and swimming

**Fig. 1:**
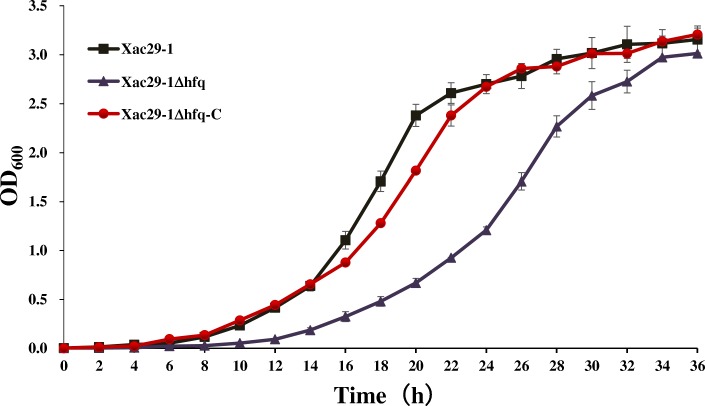
The growth curve of *Xac*29–1, *Xac*29–1Δ*hfq* and *Xac*29–1Δ*hfq-*C in NB culture medium for 36 h. The experiment was repeated three times

A mutant named *Xac*Δ*hfq* was generated by plasmid integration and confirmed by PCR and Southern blot (Additional file 1: Figure S1). Wild type *Xac*29–1, mutant *Xac*Δ*hfq* and its complemented strain *Xac*Δ*hfq*-C were cultured in NB media to examine their growth curve. As shown in Fig. 1, the loss of *hfq* led to remarkably reduced growth rate, while was restored by the complemented strain. The growth rate of *hfq* mutant was very close to wild type at stationary growth stage (36 h).

**Fig. 2:**
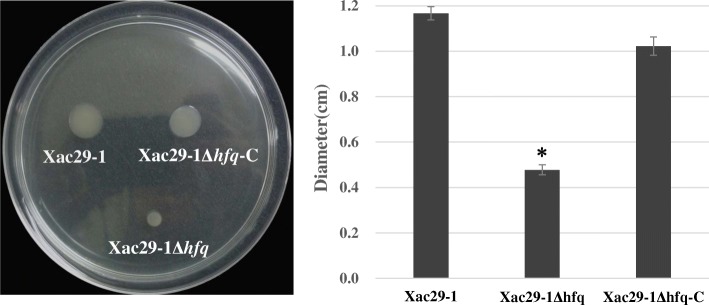
Swimming pattern of *Xac*29–1, *Xac*29–1Δ*hfq* and *Xac*29–1Δ*hfq-*C in NB medium at 48 h post inoculation. The swimming motility was measured from the diameter of each colony. The experiment was repeated three times. The asterisks in horizontal data column indicate significant differences at *P* = 0.01 by *t* test

The cell motility ability was evaluated on 0.3% (w/v) NA plates. The diameters of the motility zone derived from *hfq* mutant reduced by almost 60% when compared with the wild type. The complemented mutant strain *Xac*Δ*hfq*-C restored the motility (Fig. 2).

### *hfq* gene was involved in *Xac* biofilm formation, but did not affect *Xac* virulence

**Fig. 3:**
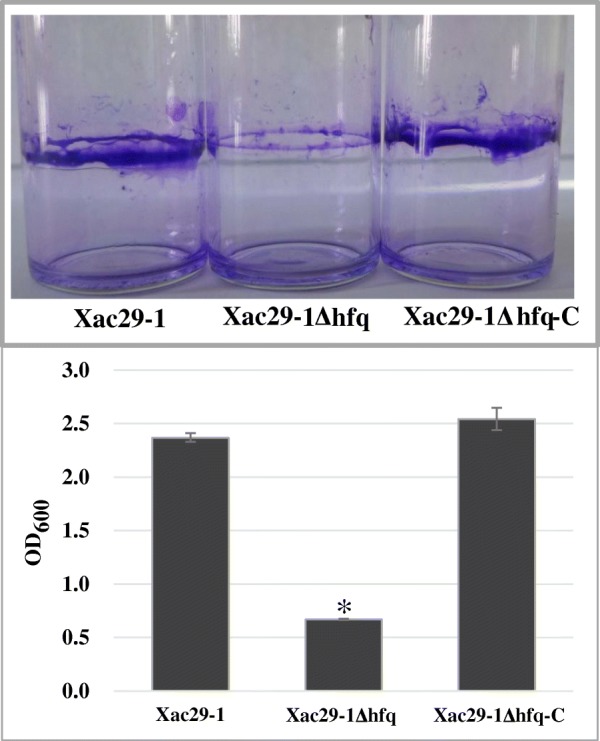
Biofilm formation of *Xac*29–1, *Xac*29–1Δ*hfq* and *Xac*29–1Δ*hfq-*C on glass bottle surfaces after 3 days incubation. The results of the biofilm formation assays were quantified by measuring the absorbance of the crystal violet stain at 600 nm. The tests were repeated three times. The asterisks in horizontal data column indicate significant differences at *P* = 0.01 by *t* test

To assess biofilm formation, the strains were grown statically in borosilicate glass tubes in NB medium for 3 days. Staining of bacterial cells with crystal violet (CV) stain showed that *Xac*29–1 and *Xac*Δ*hfq*-C produced much more biofilms of cell mass adhered to the glass surface than those produced by *Xac*Δ*hfq* strain. Accordingly, the absorbance value of crystal violet from wild type was over three times greater than that of the *hfq* mutant (Fig. 3). A cell-counting kit (CCK)-8 assay was conducted to evaulate the cell viability, and the results demonstrated that cell viability was only slightly inhibited in *hfq* mutant and no significant difference was found between *Xac*29–1 and *hfq* mutant (Data not shown).

For pathogenicity test, wound infection assay in sweet orange leaves was used. At 4 days post inoculation, all strains induced spongy-like canker symptoms, indicating that *hfq* does not contribute to the virulence of *Xac* (Additional file 2: Figure S2).

### The *hfq* mutation impairs bacterial resistance to hydrogen peroxide (H_2_O_2_) and pH

**Fig. 4:**
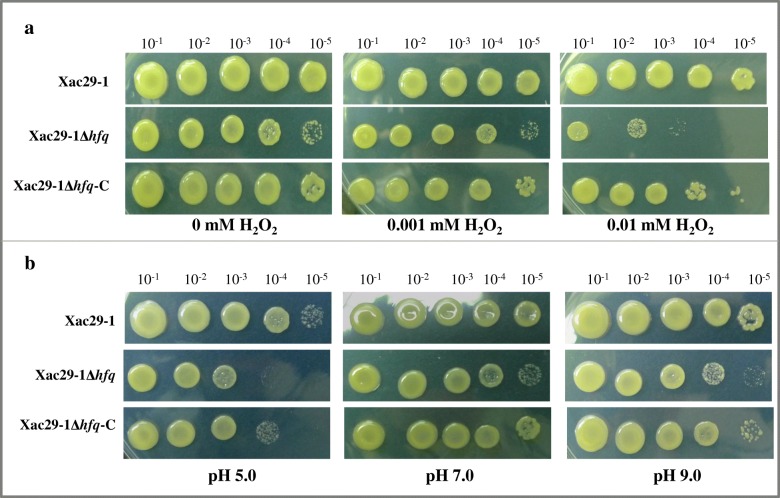
*hfq* mutations impair resistance to H_2_O_2_ and pH in *Xanthomonas axonpodis* pv. *citri*. *Xac*29–1, *Xac*29–1Δ*hfq* and *Xac*29–1Δ*hfq-*C, were grown on nutrient broth (NB) agar plates with 0 mM H_2_O_2_, 0.001 mM H_2_O_2_, or 0.01 mM H_2_O_2_ (**a**) and with pH 5.0, pH 7.0, or pH 9.0 (**b**). Three replicates for each treatment were used, and the experiment was repeated three times.

Compared with the NB medium, the growth of the *hfq* mutant almost showed no difference at the low concentration (0.001 mM) of H_2_O_2_, whereas the growth of the mutant was inhibited at 0.01 mM H_2_O_2_. The resistance of *Xac* to high and low pH was significantly affected by the mutation of *hfq* although the growth of complementation strain was also slightly affected at the high and low pH conditions (Fig. 4, Additional file 3: Figure S3). Taken together, the *hfq* mutation impairs *Xac* resistance to H_2_O_2_ and pH.

### Global RNA expression changes in *hfq* mutant of *Xac*

RNA-Seq data have been submitted to the NCBI database and the accession numbers of wild type and *hfq* mutant are PRJNA477585 and PRJNA477663, respectively. Disruption of *hfq* significantly changed expression of 746 genes. Of them, 662 genes were down-regulated and 84 genes were up-regulated. The differential expressed genes (DEGs) were enriched into different function categories through Gene ontology (GO) enrichment analysis (Additional file 3: Figure S3). The most significant GO terms in cellular component GO terms included cellular component (GO:0005575), membrane (GO:0016020), membrane part (GO:0044425) and intrinsic to membrane (GO:0031224). Besides, localization (GO:0051179) and transport (GO:0006810) in biological process GO term, receptor activity (GO:0004872) in molecular function GO term were also enriched (Additional file 4: Figure S4). Kyoto Encyclopedia of Genes and Genomes (KEGG) pathway enrichment analysis showed that most of DEGs in pathways related to bacterial chemotaxis, bacterial secretion system, two-component system, quorum sensing and flagellar assembly were repressed, while the ribosome related genes were significantly up-regulated in *hfq* mutant (Additional file 5: Table S1).

**Fig. 5:**
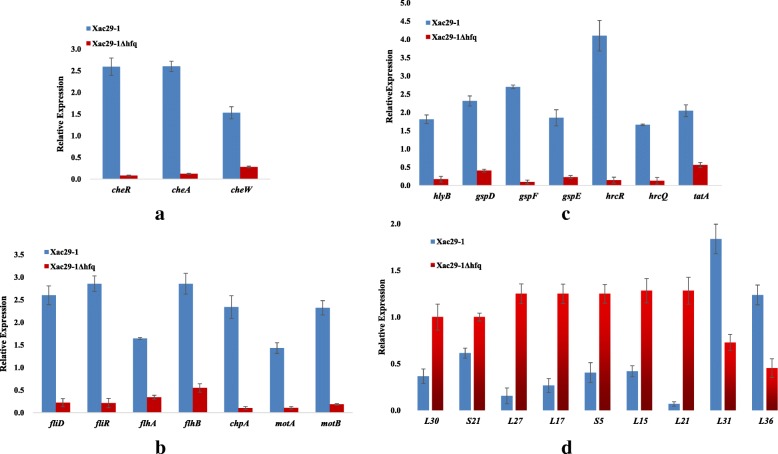
RT-qPCR of 26 selected differentially expressed genes (DEGs) related with bacterial chemotaxis (**a**), flagellar assembly (**b**), secretion system (**c**) and ribosomal protein (**d**). Three replicates for each treatment were used, and the experiment was repeated three times. Vertical bars represent standard errors

To validate the RNA-seq data, 26 genes related with chemotaxis, flagella biosynthesis, secretion system and ribosome were chosen for RT-qPCR (Fig. 5). The expression trends of 24 genes were similar with those revealed by RNA-seq results, demonstrating the reliability of RNA-seq analysis. The down-regulation expression of XAC29_11660 (50S ribosomal protein L36) and XAC29_17265 (50S ribosomal protein L31) by RT-qPCR was inconsistent with those obtained from the RNA-seq.

## Discussion

Although the inactivation of *hfq* in different bacterial species has exhibited a pleiotropic phenotype [4, 17–19], its deletion in the genus *Xanthomonas* displayed similar alterations in growth and motility [13, 14]. In this study, the mutation in *hfq* gene resulted in remarkably reduced bacterial growth rate. Additionally, the deletion of *hfq* led to a reduction of cell swimming ability by 60% and biofilm formation was reduced by almost 70%. Transcriptome and RT-qPCR analysis showed that three bacterial chemotaxis related genes, *cheR*, *cheA*, *cheW*, and seven flagella genes, *fliD*, *fliR*, *flhA*, *flhB*, *chpA*, *motA*, *motB*, were significantly repressed in the *hfq* mutant (Additional file 5: Table S1). CheA, CheW and CheR are core proteins of chemosensory pathways which are essential for motility and pathogenicity in many bacteria [20]. Transcriptome data related to chemotaxis and flagellar assembly strongly supported the biological results of the attenuation of cell motility and biofilm formation (Fig. 5, Additional file 5: Table S1).

Hfq is a common regulator of virulence in bacteria [3]. However, the virulence-related defects are not common in plant–pathogenic bacteria and only reported in a few bacteria, i.e., *Agrobacterium tumefaciens* and *Erwinia* spp. [5, 21]. Hfq significantly regulated type III secretion system and the other related pathogenicity determinants in *E.amylovora* [5]. *hfq* mutant attenuates the tumor formation and influences the virulence of *A. tumefaciens*, but the DNA-transferring type IV secretion system is not affected [21]. Transcriptome analysis showed that *hfq* significantly regulated chemotaxis, bacterial secretion systems, two-component system, quorum sensing and flagellar assembly in *Xac*. Regarding the expression profile of the genes associated with secretion systems (type I to type VI), those from type IV and type V secretion systems were unaltered, whereas two genes in type I were slighted repressed and 18 genes from type II, III, VI secretion systems were significantly decreased (over 2 folds) in *hfq* mutant (Additional file 5: Table S1). Similar with previous studies on *Xoo* and *Xcv* (13, 14), the virulence of the *hfq* mutant of *Xac* also did not affected through wound infection assay. It should be noted that virulence phenotype between *Xac* wild type and *waxcO* mutant had no difference by wound infiltration, whereas significant differences in lesion numbers were observed by spray assay [22]. Non-wound inoculation, spraying or swabbing, remains to be used to check the pathogenicity phenotype of *hfq* mutant of *Xac*, *Xoo* and *Xcv*.

It is interesting that nine ribosomal protein genes were significantly up-regulated through transcriptome data. Of the nine proteins, RpsE codes for 30S subunit ribosomal protein S5 and the other eight proteins are components of the ribosomal large subunit. It should be note that there was about four-fold expression increase for *rplU*-*rpmA*, encodes for 50S subunit r-proteins L21 and L27, respectively (Additional file 5: Table S1). RT-qPCR also proved the high expression of the operon genes in *hfq* mutant (Fig. 5). L27, consisting of a C-terminal β-sandwich domain and a long N-terminal arm, plays a key role in tRNA substrate stabilization during the peptidyl transfer reaction [23, 24]. *RpsE* has been implicated in tRNA selection and translation fidelity in *E. coli* [25]. Although it remains unclear how Hfq affects these ribosomal proteins of *Xac*, the expression differences of ribosomal proteins, probably resulted in reduced translation accuracy, might also play certain roles in the pleiotropic defects of *hfq* mutants.

Many sRNA candidates in genus *Xanthomonas* have been generated by high-throughput transcriptome sequencing approaches [13–15]. Of these sRNAs, 44 sRNAs were so far experimentally verified in *Xoo*, *Xcv* and *Xcc* [26]. It should be noted that the accumulation and activity of only six sRNAs were closely related with *hfq* whereas some sRNAs involved in virulence, i.e. sX12 and sX13, were assumed to act Hfq-independent (14, 26). It might partially explain why the *hfq* mutant strain of *Xanthomonas* spp. was not altered in the induction of virulence phenotype. So far, the function of Hfq is still obscure in *Xanthomonas* spp. and its physiological roles and RNA-binding capability is needed to be further addressed. This study is a good starting point for identifying regulatory sRNAs and genes controlled by Hfq-sRNA interactions in *Xac*.

## Conclusions

In this study, biological analyses of the *hfq* mutant clearly point toward the requirement for Hfq function in multiple biological processes in *Xac*, i.e. motility, biofilm formation and stress response. RNA-seq data showed that 746 genes were regulated by *hfq* gene, reflecting its global regulation role in *Xac*. In particular, the expression of genes associated with bacterial chemotaxis and flagellar assembly were significantly down-regulated in the *hfq* mutant, consistent with the reduction of swimming and biofilm formation.

## Methods

### Bacterial strains, plasmids and growth conditions

The bacterial strains and plasmids used in this work are listed in Table 1. *Xanthomonas* strains were grown at 28 °C in nutrient broth (NB) medium or on NA (NB with 1.5% Agar). *Escherichia coli* strains were cultured at 37 °C in Luria–Bertani (LB) medium or on LA (LB with 1.5% Agar). When required, the antibiotics kanamycin (50 μg/ml) and gentamycin (10 μg/mL) were added to the growth media.

**Table 1:** Strains, plasmids and primers used in this study

Strains, plasmids and primers	Feature	Source
Strains		
*Xac*29–1	G^−^, wt	Ye et al. (2013)
*Xac*29–1Δ*hfq*	G^−^, Δ*hfq*	This study
*Xac* 9-1Δ*hfq*-C	G^−^, *hfq*^+^	This study
JM109	G^−^	Takara
Plasmids		
pEASY-T1	Kan^+^/Amp^+^	TransGen
pK18mob*SacB*	Kan^+^, *SacB*	Zou et al. (2011)
pK18mob*SacB*-Δ*hfq*	Kan^+^, *SacB, hfq*^+^	This study
pBBR1MCS-5	Gm^+^	Zou et al. (2011)
Primers		
*hfq*-F	5′-GCTTCAGGCGTGTAACATCC-3’	This study
*hfq*-R	5′-GCGAACTCCTCCAACACATC-3’	This study
*hfq*-up-F	5′-TGGGATCCCGCGTGTTGAAGGTGGTATT-3’	This study
*hfq*-up-R	5′-TGGGTACCCGAAAAATCCTCTTCATTATTGT-3’	This study
*hfq*-down-F	5′-TGGGTACCCGGAGTAGTGCGTGTTTGATC-3’	This study
*hfq*-down-R	5′-GCTCTAGAAAGCCTCTGCACCGGTCAACA-3’	This study
*hfq*-F1	ATGGCTAAGGGGCAATCTTTAC	This study
*hfq*-R1	ACCGATCAAACACGCACTACT	This study

### Construction of the *hfq* deletion mutant and its complemented strain

The *hfq* mutant was generated from *Xac*29–1 wild type strain by allelic homologous recombination. Briefly, two *hfq* flanking regions were amplified by PCR using the primer pairs up F/R and down F/R (Table 1). The PCR products of upstream and downstream were digested with *Bam*HI/*Kpn*I and *Kpn*I/*Xba*I, respectively. The digested fragments were ligated into the suicide vector pK18mob*SacB* to obtain the recombinant plasmid pK18mob*Sac*B-Δ*hfq*. The plasmid was transformed into wild type strain *Xac*29–1 by electroporation. The *hfq* mutant, named *Xac*29–1Δ*hfq*, was obtained after two recombination events and confirmed by PCR and Southern blotting.

To complement the *hfq* mutant, DNA fragment containing the entire *hfq* gene and its upstream promoter was amplified. The amplified fragment was digested with HindIII/*Eco*RI enzyme and cloned into HindIII/*Eco*RI-digested pBBR1MCS-5 [27], resulting in pBBR1MCS-5 + *hfq* plasmid. The complementary plasmid was transformed and one complemented mutant strain, named *Xac*29–1Δ*hfq-*C, was selected on NA plate with Gentamycin resistance.

### Determination of growth curve

Pellets of *Xac*29–1, *Xac*29–1Δ*hfq* and *Xac*29–1Δ*hfq*-C strains were cultured in NB medium and adjusted to an OD_600_ =0.6 and then sub-cultured (1100) in fresh NB for 36 h. The OD_600_ values were tested after every 2 h post sub-culturing. All the experiments were repeated at least three times.

### Motility assay

To test cell motility, all strains were grown overnight in NB medium and adjusted to an OD_600_ =0.6 [10^8^ colony-forming units (cfu)/mL]. 2 μL of each cell sample was dropped to 0.3% agar NA plates for the swimming motility tests [18]. The diameters of each colony were measured after 48 h of incubation, and the resulting values were taken to indicate the bacterial motility.

### Biofilm formation assay

Biofilms that formed on polystyrene and glass surfaces were examined as previously described [28]. Briefly, 5 μL of each adjusted cell sample was transferred to a glass bottle containing 5 mL fresh NB medium and stationary incubated at 28 °C for 3 days. After the medium removal and washing, bacteria were stained with 5 mL crystal violet for 5 min. Excess crystal violet stain was removed to observe a circle of purple material formed on the glass bottle. The crystal violet dye was solubilized by addition of 5 mL organic solvent (anhydrous ethanol: acetone = 70:30, v/v), then crystal violet was quantified by measuring absorbance at 600 nm. All the experiments were repeated three times and the average for each strain was checked by *t*-test.

### Cell viability assay

A CCK-8 assay was used to determine the cell viability of *Xac*29–1, *Xac*29–1Δ*hfq* and *Xac*29–1Δ*hfq*-C strains incubated at 28 °C for 3 days. 100 μL of each cell sample and 10 μL CCK-8 (BioDee Biotechnology, Beijing, China) were plated into 96-well plates and incubated at 37 °C for 4 h. Then the absorbance was measured at 450 nm wavelength. Each group had three wells and all experiments were repeated three times.

### Stress resistance assays

The resistance assay against H_2_O_2_ was performed as described previously [29] with minor modifications. *Xac*29–1, *Xac*29–1Δ*hfq* and *Xac*29–1Δ*hfq*-C strains were cultured in NB medium and adjusted to an OD_600_ =0.6. H_2_O_2_ with a concentration of 0.1 mM and 0.001 mM, were supplemented to the bacterial suspension and incubated at 28 °C for 10 min with shaking, respectively. The challenged bacterial cells were diluted by 5 gradients (10^− 1^, 10^− 2^, 10^− 3^, 10^− 4^, 10^− 5^), 2 μL of each cell sample was dropped on NA plates respectively and stationary incubated at 28 °C for 3 days.

pH stress testing was similar with that of H_2_O_2_ testing. The adjusted bacterial population was diluted by 5 serial dilutions (10^− 1^, 10^− 2^, 10^− 3^, 10^− 4^, 10^− 5^). 2 μL of each cell sample was dropped on NA plates with pH of 5.0, 6.0, 7.0, 8.0, 9.0, respectively.

### Pathogenicity assay

Strains were cultured and adjusted to an OD_600_ =0.6. The full expanded leaves of pineapple sweet orange (*Citrus sinensis*) were used as host materials. Five wounds were produced on the back of the leaves with a needle. 10 μL of each cell suspension were then placed on the wounds. Disease symptoms were observed and photographed 2 days post inoculation [30]. Each test was repeated at least three times.

### RNA extraction, library preparation and RNA sequencing

Bacterial cultures from wild-type and *hfq* mutant strains were collected in the middle exponential stage (OD_600_ = 0.6–0.8). RNAs were extracted using the RNA prep pure Cell/Bacteria Kit (Tiangen Biotech, Beijing, China). Strand-specific RNA-seq libraries were generated using NEBNext® Ultra™ Directional RNA Library Prep Kit for Illumina® (NEB, USA). After cluster generation, the library preparations were sequenced on an Illumina Hiseq platform (Illumina, CA, USA) in Novogene, Tianjing.

### Transcriptome data analysis

After cleaning the raw reads, we mapped the clean reads to the complete genome *Xac*29–1 strain (CP004399.1, CP004402.1, CP004401.1 and CP004400.1) using Bowtie 2 [31] and then calculated gene expression level with RSEM [32]. Differentially expression analysis was performed using the DESeq R package (1.18.0). The *p*-values were adjusted for the false discovery rate (FDR). Genes with an adjusted *p-*value < 0.05 and |log_2_(Fold Change)| > 1 were considered to be differentially expressed genes at a statistically significant level. A *t*-test was performed on log_2_-transformed data to identify the genes with significant changes in expression between wild type and mutant strains. Gene Ontology (GO) terms with corrected *P* value ≤0.05 were considered significantly enriched by differentially expressed genes.

### RT-qPCR

To assess the RNA-seq quality, the same set RNAs for RNA-seq were subjected to a two-step RT-qPCR assay with Bio-Rad iQ5 Real Time PCR System (Bio-Rad, CA, USA) using SYBR green RT-PCR kit (Promega). 26 genes related with bacterial chemotaxis, flagellar assembly, bacterial secretion and ribosome were chosen for RT-qPCR (Additional file 6: Table S2). The 16S rRNA gene was used as an endogenous control. The relative fold change in target gene expression was calculated using the 2^–ΔΔCT^ method [33].

## Additional files

Additional file 1: **Figure S1.** PCR and southern blotting confirmation of the *hfq* mutant. (A) The gene deletion scheme. The 480-bp (amplified by *hfq*-up-F/R) (Table 1) and 680-bp (amplified by *hfq*-down-F/R) (Table 1) DNA fragments were used as the 5′and 3′fragments for homologous recombination, respectively. The 278-bp DNA fragment of *hfq* gene was deleted in the *hfq* mutant. The *hfq*-F/R primer (Table 1) was used for molecular confirmation of the *hfq* mutant. If the 278-bp fragment of *hfq* gene was successfully deleted, a 195-bp DNA fragment would be amplified from the mutant. (B) PCR confirmation of the *hfq* mutant. M, Mark; 1–6, *hfq* deletion mutant; 7–8, *Xac*29–1 wild type strain; 9, pK18mob*SacB*-Δ*hfq*, positive control; 10, H_2_O, negative control. (C) Southern blotting analysis of the *hfq* deletion mutant. The 794-bp fragment was used as the probe for Southern blotting. A 1.8-kb DNA fragment was detected in the *Xac*29–1 wild-type strain (lane 3), whereas only an approximately1.5-kb fragment was obtained in the *hfq* deletion mutant (lane 1 and 2) owing to the deletion of the 279-bp fragment. (PPTX 61 kb)

Additional file 2: **Figure S2.** The pathogenicity test of *Xac*29–1 strain, *hfq* mutant and complementary strain by wound infection on detached citrus leaves. Each test was repeated at least three times. (PPTX 116 kb)

Additional file 3: **Figure S3.** *hfq* mutations impair resistance to pH in *Xanthomonas axonpodis* pv. *citri* (repeat experiment). *Xac*29–1, *Xac*29–1Δ*hfq* and *Xac*29–1Δ*hfq-*C, were grown on nutrient broth (NB) agar plates with pH 5.0, pH 7.0, or pH 9.0. (PPTX 801 kb)

Additional file 4: **Figure S4.** Gene ontology (GO) enrichment analysis of differentially expressed genes (DEGs) of *Xac*29–1 wild-type strain compared with *hfq* mutant. Up, up-regulation; Down, own-regulation. (PPTX 63 kb)

Additional file 5: **Table S1.** List of the differentially expressed genes in bacterial chemotaxis, two-component system, secretion system, quorum sensing, flagellar assembly and ribosome. (DOCX 20 kb)

Additional file 6: **Table S2.** The primers used for RT-qPCR validation. (DOCX 17 kb)

## Abbreviations

CV: Crystal violet
DEG: Differentially expressed gene
FDR: False discovery rate
GO: Gene Ontology
LB: Luria–Bertani
NA: Nutrient agar
NB: Nutrient broth
OD: Optical density
RNA-Seq: RNA sequencing
RT-PCR: Reverse transcription- polymerase chain reaction
sRNA: small non-coding RNA
