# Supplementary figures and images for: Biological and transcriptomic studies reveal hfq is required for swimming, biofilm formation and stress response in Xanthomonas axonpodis pv. citri
Source: BMC Microbiol. 2019 May 22;19:103. doi: 10.1186/s12866-019-1476-9 (PMC6530196; doi:10.1186/s12866-019-1476-9)

## Slide 1
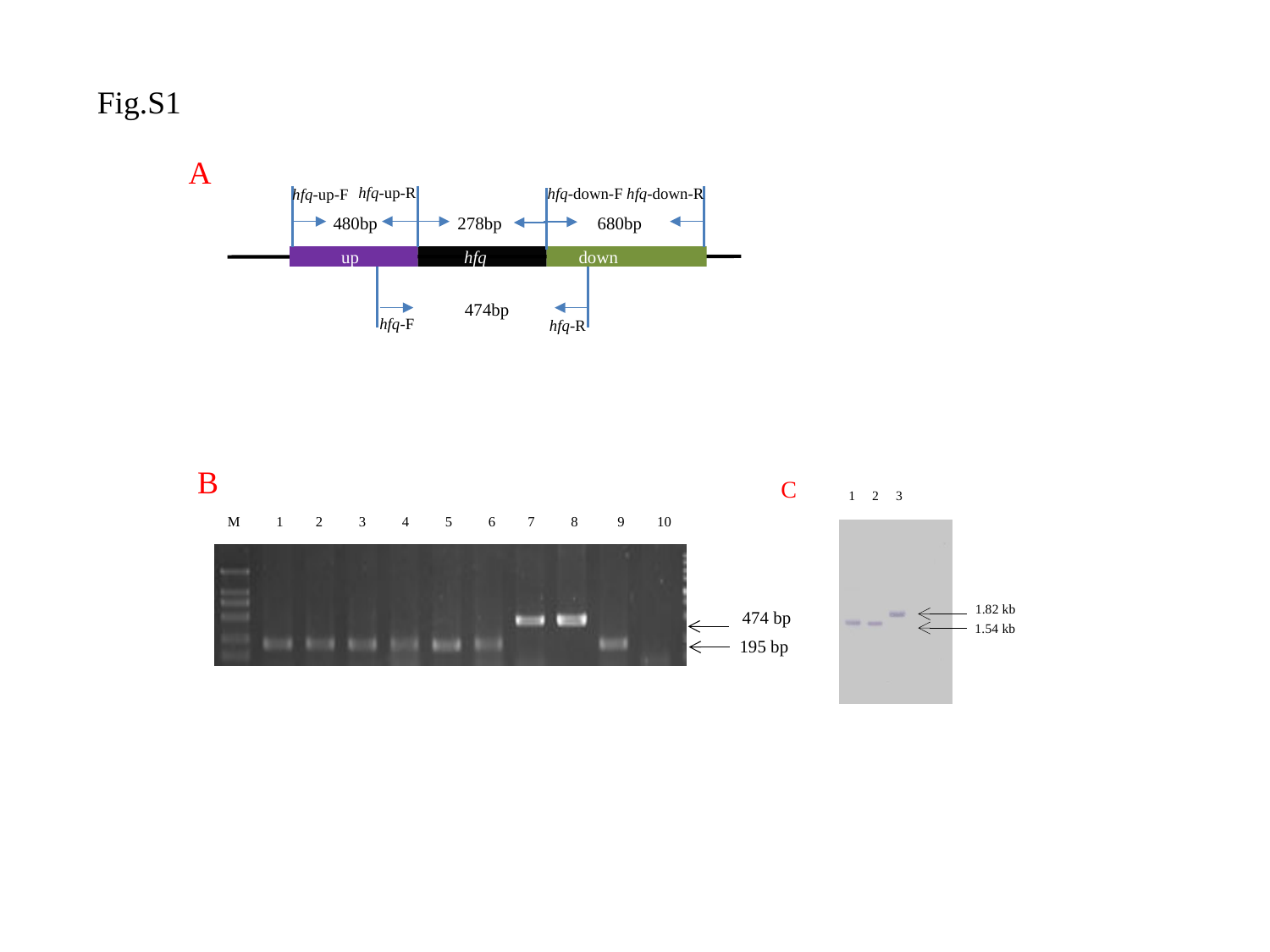

Fig.S1
A
hfq-up-R
hfq-down-R
hfq-down-F
hfq-up-F
278bp
up
hfq
down
hfq-F
hfq-R
480bp
680bp
474bp
B
C
1 2 3
M 1 2 3 4 5 6 7 8 9 10
474 bp
195 bp
1.82 kb
1.54 kb

Supplement: Supplementary file 1 — Figure S1. PCR and southern blotting confirmation of the hfq mutant. (A) The gene deletion scheme. The 480-bp (amplified by hfq-up-F/R) (Table 1) and 680-bp (amplified by hfq-down-F/R) (Table 1) DNA fragments were used as the 5′and 3′fragments for homologous recombination, respectively. The 278-bp DNA fragment of hfq gene was deleted in the hfq mutant. The hfq-F/R primer (Table 1) was used for molecular confirmation of the hfq mutant. If the 278-bp fragment of hfq gene was successfully deleted, a 195-bp DNA fragment would be amplified from the mutant. (B) PCR confirmation of the hfq mutant. M, Mark; 1–6, hfq deletion mutant; 7–8, Xac29–1 wild type strain; 9, pK18mobSacB-Δhfq, positive control; 10, H2O, negative control. (C) Southern blotting analysis of the hfq deletion mutant. The 794-bp fragment was used as the probe for Southern blotting. A 1.8-kb DNA fragment was detected in the Xac29–1 wild-type strain (lane 3), whereas only an approximately1.5-kb fragment was obtained in the hfq deletion mutant (lane 1 and 2) owing to the deletion of the 279-bp fragment. (PPTX 61 kb) [file 12866_2019_1476_MOESM1_ESM.pptx]

## Slide 1
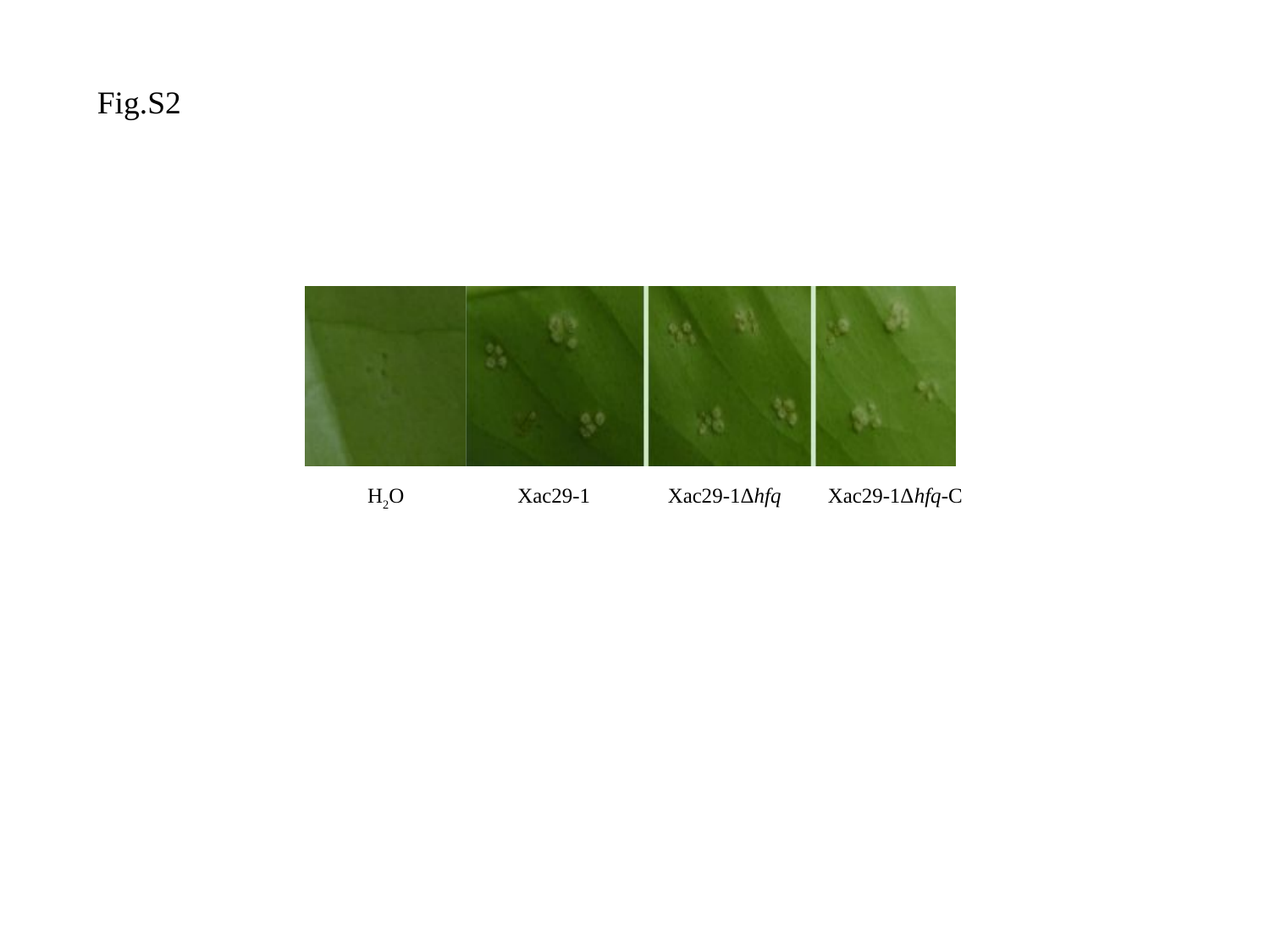

Fig.S2
H2O
Xac29-1
Xac29-1Δhfq
Xac29-1Δhfq-C

Supplement: Supplementary file 2 — Figure S2. The pathogenicity test of Xac29–1 strain, hfq mutant and complementary strain by wound infection on detached citrus leaves. Each test was repeated at least three times. (PPTX 116 kb) [file 12866_2019_1476_MOESM2_ESM.pptx]

## Slide 1
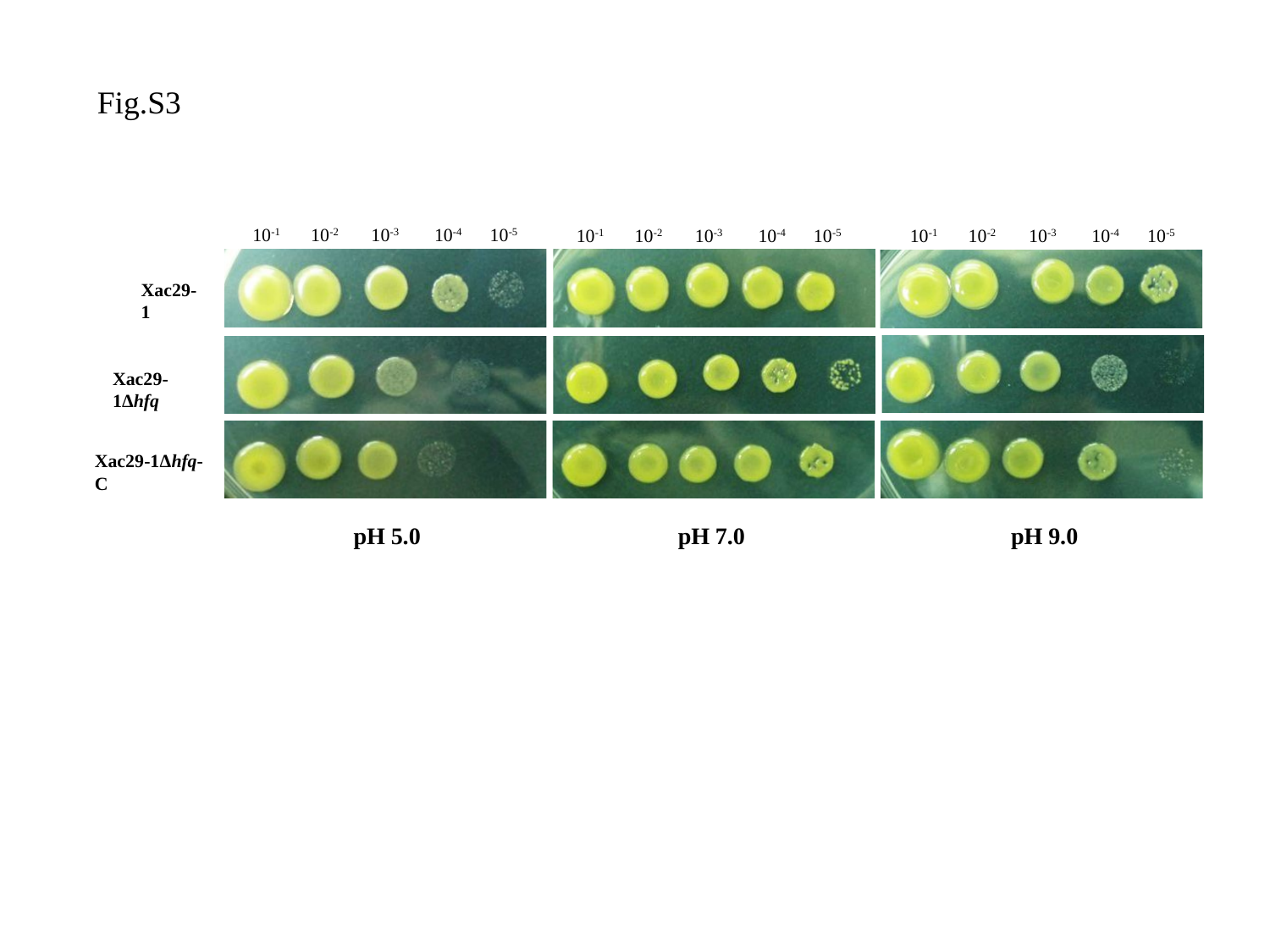

Fig.S3
10-1 10-2 10-3 10-4 10-5
10-1 10-2 10-3 10-4 10-5
10-1 10-2 10-3 10-4 10-5
Xac29-1
Xac29-1Δhfq
Xac29-1Δhfq-C
pH 5.0
pH 7.0
pH 9.0

Supplement: Supplementary file 3 — Figure S3. hfq mutations impair resistance to pH in Xanthomonas axonpodis pv. citri (repeat experiment). Xac29–1, Xac29–1Δhfq and Xac29–1Δhfq-C, were grown on nutrient broth (NB) agar plates with pH 5.0, pH 7.0, or pH 9.0. (PPTX 801 kb) [file 12866_2019_1476_MOESM3_ESM.pptx]

## Slide 1
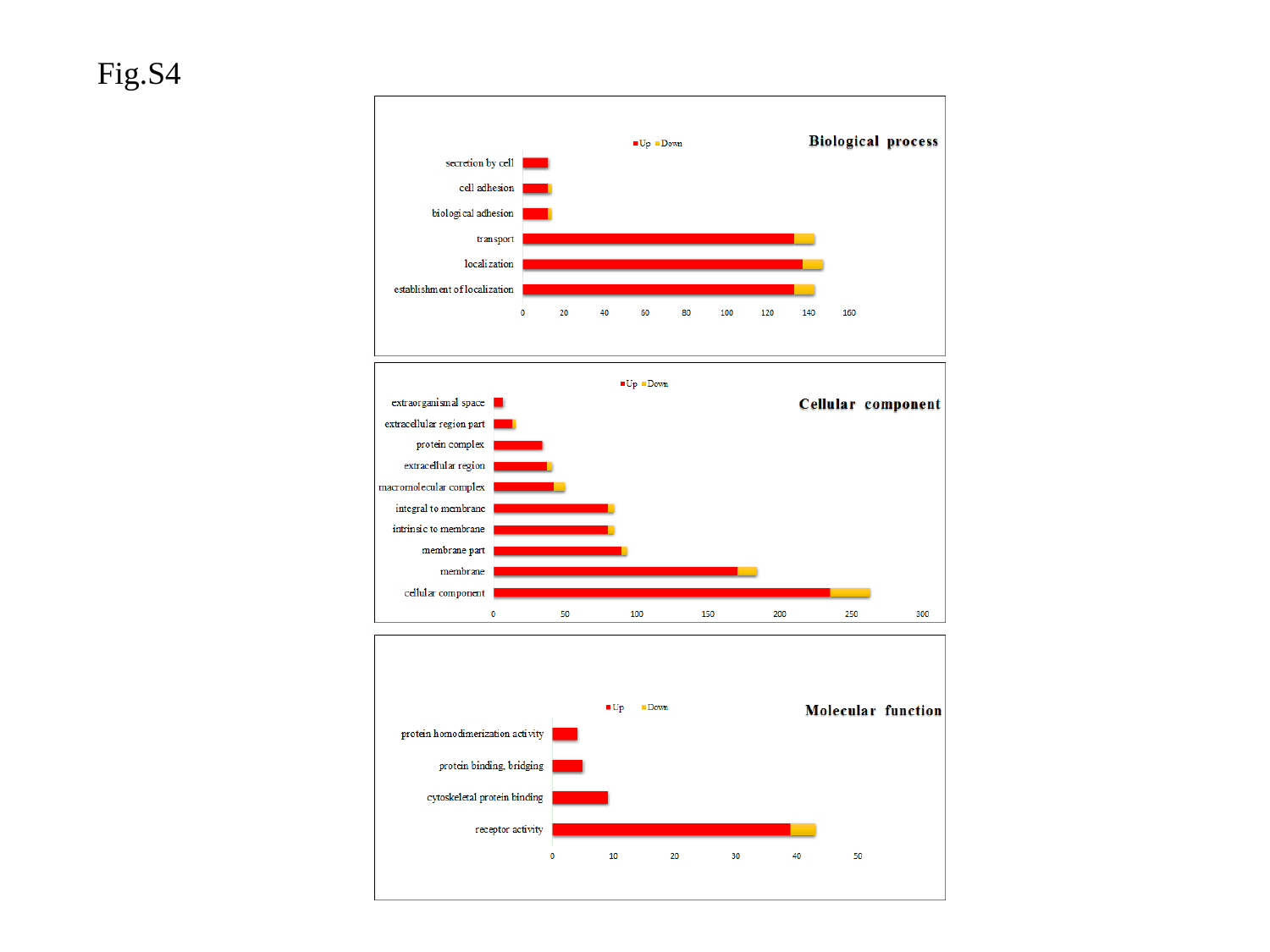

Fig.S4

Supplement: Supplementary file 4 — Figure S4. Gene ontology (GO) enrichment analysis of differentially expressed genes (DEGs) of Xac29–1 wild-type strain compared with hfq mutant. Up, up-regulation; Down, own-regulation. (PPTX 63 kb) [file 12866_2019_1476_MOESM4_ESM.pptx]
