# Supplementary material for: Biological and transcriptomic studies reveal hfq is required for swimming, biofilm formation and stress response in Xanthomonas axonpodis pv. citri
Source: BMC Microbiol. 2019 May 22;19:103. doi: 10.1186/s12866-019-1476-9 (PMC6530196; doi:10.1186/s12866-019-1476-9)
Supplement: Supplementary file 5 — Table S1. List of the differentially expressed genes in bacterial chemotaxis, two-component system, secretion system, quorum sensing, flagellar assembly and ribosome. (DOCX 20 kb) [file 12866_2019_1476_MOESM5_ESM.docx]

Table S1 List of the differentially expressed genes in bacterial chemotaxis, two-component system, secretion system, quorum sensing, flagellar assembly and ribosome.

| Gene_ID | log2.Fold_change. | description |
| --- | --- | --- |
| **Bacterial chemotaxis** | |  |
| XAC29_09540 | 1.2311 | chemotaxis protein methyltransferase |
| XAC29_09545 | 1.2382 | methyl-accepting chemotaxis protein |
| XAC29_09565 | 1.0868 | methyl-accepting chemotaxis protein |
| XAC29_09580 | 1.0945 | methyl-accepting chemotaxis protein |
| XAC29_09595 | 1.4018 | chemotaxis protein |
| XAC29_09605 | 1.6118 | chemotaxis protein |
| XAC29_09620 | 1.1192 | chemotaxis protein |
| XAC29_09670 | 1.1224 | flagellar motor protein |
| XAC29_12415 | 1.7351 | chemotaxis protein |
| XAC29_12420 | 1.8877 | chemotaxis protein |
| XAC29_14610 | 1.7273 | chemotaxis histidine protein kinase |
| XAC29_14615 | 1.8221 | chemotaxis protein |
| XAC29_14630 | 1.7403 | response regulator for chemotaxis |
| XAC29_16350 | 1.6645 | chemotaxis protein |
| XAC29_16680 | 1.1294 | chemotaxis transducer |
| XAC29_18795 | 1.0645 | flagellar motor protein MotA |
| XAC29_18800 | 1.201 | flagellar motor protein MotB |
| **Two-component system** | |  |
| XAC29_01555 | 2.3468 | transcriptional regulator |
| XAC29_01680 | 3.0328 | membrane fusion protein |
| XAC29_01685 | 2.2835 | multidrug efflux transporter |
| XAC29_01690 | 2.3102 | hypothetical protein |
| XAC29_03155 | 1.1121 | two-component system sensor protein |
| XAC29_03860 | 1.5576 | potassium-transporting ATPase subunit B |
| XAC29_03865 | 1.5943 | potassium-transporting ATPase subunit C |
| XAC29_08780 | -1.2235 | carbon storage regulator |
| XAC29_09540 | 3.2963 | chemotaxis protein methyltransferase |
| XAC29_09545 | 3.5789 | methyl-accepting chemotaxis protein |
| XAC29_09565 | 2.6129 | methyl-accepting chemotaxis protein |
| XAC29_09580 | 2.4225 | methyl-accepting chemotaxis protein |
| XAC29_09595 | 1.6615 | chemotaxis protein |
| XAC29_09605 | 1.5943 | chemotaxis protein |
| XAC29_09620 | 2.7288 | chemotaxis protein |
| XAC29_09670 | 2.7086 | flagellar motor protein |
| XAC29_11410 | 2.2125 | PilL protein |
| XAC29_12415 | 1.7351 | chemotaxis protein |
| XAC29_12420 | 2.7809 | chemotaxis protein |
| XAC29_14610 | 1.7273 | chemotaxis histidine protein kinase |
| XAC29_14615 | 2.9096 | chemotaxis protein |
| XAC29_14630 | 2.9509 | response regulator for chemotaxis |
| XAC29_16350 | 2.0145 | chemotaxis protein |
| XAC29_18795 | 3.017 | flagellar motor protein MotA |
| XAC29_18980 | 1.3593 | cyanide insensitive terminal oxidase |
| XAC29_16525 | -1.0193 | fimbrillin |
| XAC29_16680 | 1.1294 | chemotaxis transducer |
| XAC29_20975 | 2.2145 | alkaline phosphatase |
| XAC29_20980 | 2.2548 | alkaline phosphatase |
| **Bacterial secretion system** | |  |
| XAC29_02060 | 3.4372 | type III secretion system protein YscR |
| XAC29_02065 | 3.1906 | HrcQ protein (T3SS) |
| XAC29_02075 | 2.2249 | HrcV protein (T3SS) |
| XAC29_02080 | 2.5007 | type III secretion system protein HrcU |
| XAC29_02105 | 2.1467 | type III secretion system protein HrpB |
| XAC29_02110 | 2.3281 | type III secretion system ATPase |
| XAC29_02120 | 2.1745 | HrcT protein (T3SS) |
| XAC29_03530 | 2.9905 | type II secretion system protein C |
| XAC29_03535 | 2.8451 | type II secretion system protein D |
| XAC29_03540 | 3.0092 | type II secretion system protein E |
| XAC29_03545 | 3.2013 | type II secretion system protein F |
| XAC29_03565 | 2.4745 | type II secretion system protein J |
| XAC29_03570 | 2.9339 | type II secretion system protein K |
| XAC29_03575 | 2.5983 | type II secretion system protein L |
| XAC29_11145 | 1.0039 | hemolysin secretion protein D (T1SS) |
| XAC29_11150 | 1.9333 | hemolysin secretion protein B(T1SS) |
| XAC29_20745 | 2.2874 | hypothetical protein (T6SS) |
| XAC29_20750 | 2.7937 | hypothetical protein (T6SS) |
| XAC29_20835 | 2.6485 | chaperone ClpB (T6SS) |
| XAC29_21270 | -1.0126 | twin arginine translocase protein A |
| XAC29_20770 | 2.0126 | hypothetical protein (T6SS) |
| **Quorum sensing** |  |  |
| XAC29_04360 | 2.0654 | ABC transporter oligopeptide-binding protein |
| XAC29_04365 | 2.7189 | ABC transporter permease |
| XAC29_04370 | 2.5314 | ABC transporter permease |
| XAC29_04380 | 2.848 | ABC transporter ATP-binding protein |
| XAC29_04725 | 1.9359 | extracellular protease |
| XAC29_04730 | 1.9961 | extracellular protease |
| XAC29_05180 | 2.4224 | non-hemolytic phospholipase C |
| XAC29_12025 | 1.6252 | pectate lyase |
| XAC29_15180 | 1.4181 | pectate lyase II |
| XAC29_16080 | 2.0771 | phospholipase C |
| XAC29_17950 | -1.0618 | hypothetical protein |
| XAC29_18060 | 2.801 | serine protease |
| **Flagellar assembly** | |  |
| XAC29_09670 | 1.1224 | flagellar motor protein |
| XAC29_09800 | 1.3175 | flagellar biosynthesis protein FlhA |
| XAC29_09805 | 1.0745 | flagellar biosynthesis protein FlhB |
| XAC29_09825 | 1.7382 | flagellar biosynthetic protein FliR |
| XAC29_09980 | 1.466 | flagellar protein |
| XAC29_09985 | 1.1894 | flagellar protein |
| XAC29_10065 | 1.5014 | flagellar basal body P-ring biosynthesis protein FlgA |
| XAC29_18795 | 1.0645 | flagellar motor protein MotA |
| XAC29_18800 | 1.201 | flagellar motor protein MotB |
| **Ribosome** |  |  |
| XAC29_05000 | -1.8796 | 30S ribosomal protein S5 |
| XAC29_05005 | -2.2027 | 50S ribosomal protein L30 |
| XAC29_05010 | -1.9011 | 50S ribosomal protein L15 |
| XAC29_05040 | -2.4169 | 50S ribosomal protein L17 |
| XAC29_06275 | -4.4204 | 50S ribosomal protein L21 |
| XAC29_06280 | -4.433 | 50S ribosomal protein L27 |
| XAC29_11660 | -1.8535 | 50S ribosomal protein L36 |
| XAC29_17265 | -1.1408 | 50S ribosomal protein L31 type B |
| XAC29_19680 | -1.0442 | 30S ribosomal protein S21 |
