# Supplementary material for: Biological and transcriptomic studies reveal hfq is required for swimming, biofilm formation and stress response in Xanthomonas axonpodis pv. citri
Source: BMC Microbiol. 2019 May 22;19:103. doi: 10.1186/s12866-019-1476-9 (PMC6530196; doi:10.1186/s12866-019-1476-9)
Supplement: Supplementary file 6 — Table S2. The primers used for RT-qPCR validation. (DOCX 17 kb) [file 12866_2019_1476_MOESM6_ESM.docx]

Table S2 The primers used for RT-qPCR validation.

| Gene-ID | Primers | sequence（5’→3’） | Description |  |
| --- | --- | --- | --- | --- |
| (XAC29-14630) *cheR* | cheR-F | CTTCCAGCGCTTCATCTTC | Response regulator for chemotaxis | |
|  | cheR-R | ACTGCGTATAGGTACTGAAC |  |  |
| (XAC29-14610) *cheA* | cheA-F | GCCATTTTCCGCGCCGCACAC | Chemotaxis histidine protein kinase | |
|  | cheA-R | CCCGATCAGTTCCGAGCTCA |  |  |
| (XAC29-12415)  *cheW* | cheW-F | TCGCCGCGCAATATCTGACCT | Chemotaxis protein cheW | |
|  | cheW-R | CGCACGCAATCCGGCATCGAC |  |  |
| (XAC29-09985) *fliD* | fliD-F | AGCAAGTTCAGCGGCACCCT | Flagellar protein FliD | |
|  | fliD-R | GAATTGGGCGGTGTAGCGGTC |  |  |
| (XAC29-09825) *fliR* | fliR-F | AGTCCACCGGCCTGTCGTTC | Flagellar biosynthetic protein FliR | |
|  | fliR-R | ATCACCGCCAGATGCCCGTTG |  |  |
| (XAC29-09800) *flhA* | flhA-F | CATCGACGCCGACCTCAACGC | Flagellar biosynthesis protein FlhA | |
|  | flhA-R | CGCCGTCCATCGCACCGTA |  |  |
| (XAC29-09805) *flhB* | flhB-F | ACCAAGCTCAATCCCGCCAAC | Flagellar biosynthesis protein FlhB | |
|  | flhB-R | CCCCTGGAAATGCAGAAGCT |  |  |
| (XAC29-11410) *chpA* | chpA-F | AAGCCCATCGAGAGGTCTGC | PilL protein chpA | |
|  | chpA-R | TCATGGCAGCTTCTCCGAG |  |  |
| (XAC29-09670) *motA* | motA-F | AGACCAATTGCCGCGTCAGGA | Flagellar motor protein MotA | |
|  | motA-R | CCCATGCCCTCGAATACCTT |  |  |
| (XAC29-18800) *motB* | motB-F | ACAAGACCGATCCGCAGAACCC | Flagellar motor protein MotB | |
|  | motB-R | CGCTCAGATCCGGCACCT |  |  |
| (XAC29-11150) *hlyB* | hlyB-F | CGAGGCCATCATCCAGCGCAAC | Hemolysin secretion protein B | |
|  | hlyB-R | TGCGCCCCTTGTCCATCACGAT |  |  |
| (XAC29-03535) *gspD* | gspD-F | ACCTGTTGTCCACGCCCTCGAT | Type II secretion system protein D | |
|  | gspD-R | TGGTGCGGAATGGGTTGTCGTT |  |  |
| (XAC29-03545) *gspF* | gspF-F | AGTTTGATTACACCGTGCTCG | Type II secretion system protein F | |
|  | gspF-R | TTCCACACGCACCGGCACCCATT |  |  |
| (XAC29-03540) *gspE* | gspE-F | ACGTGCATCTGGAATCCTACGAA | Type II secretion system protein E | |
|  | gspE-R | TATCCAGCCGCGCCATCACCT |  |  |
| (XAC29-02060) *hrcR* | hrcR-F | CCGCCGAACATGGTCCTCA | Type III secretion system protein YscR | |
|  | hrcR-R | CACGGCTGTTATCGGACCC |  |  |
| (XAC29-02065) *hrcQ* | hrcQ-F | CCCACCATGCAGCACGATAC | HrcQ protein | |
|  | hrcQ-R | CACCGACAACTCCAGGATCT |  |  |
| (XAC29-21270)  *tatA* | tatA-F | CGGTTTCAGCATTTGGCACT | Twin arginine translocase protein A | |
|  | tatA-R | CGTCGTGCATGCCTTTCTTGA |  |  |
| (XAC29-06275)  *L21* | L21-F1 | CGCGTGGAAAAGCTCGAAGTC | Large subunit ribosomal protein L21 | |
|  | L21-R1 | CTTGATGATGCGCACCTTGTC |  |  |
| (XAC29-05000)  *S5* | S5-F1 | CCGGTCGCCATCCAAAAGTCG | Small subunit ribosomal protein S5 | |
|  | S5-R1 | CTTCCAGCACAGCGCGCAT |  |  |
| (XAC29-19680)  *S21* | S21-F1 | TCCGTCGTTTCAAGCGCACCT | Small subunit ribosomal protein S21 | |
|  | S21-R1 | TTACGCTCCTGGGTCGGCTTT |  |  |
| (XAC29-05010)  *L15* | L15-F1 | CCCGTACCGAGCGCACCC | Large subunit ribosomal protein L15 | |
|  | L15-R1 | ATCTTGCCGCCACCCTTACGAG |  |  |
| (XAC29-11660)  *L36* | L36-F1 | CTGTCCTCCCTGAAGTCTGCGA | Large subunit ribosomal protein L36 | |
|  | L36-R1 | CCTTGAAGCGCGGGTTCGACT |  |  |
| (XAC29-05005)  *L30* | L30-F1 | AGGACACCAACAAGACCGTCA | Large subunit ribosomal protein L30 | |
|  | L30-R1 | CCTTCAGTTCACGCACATC |  |  |
| (XAC29-05040)  *L17* | L17-F1 | TCAACCGTACCAGCGCACACC | Large subunit ribosomal protein L17 | |
|  | L17-R1 | CGAGCAAATGCCAGACGACGAT |  |  |
| (XAC29-17265)  *L31* | L31-F1 | CAAGATTCTGACCCGTTCCAC | Large subunit ribosomal protein L31 | |
|  | L31-R1 | TCACCTTGTGCTTGCCGGTA |  |  |
| (XAC29-06280)  *L27* | L27-F1 | GAAGTACCTCGGCGTGAAGAT | Large subunit ribosomal protein L27 | |
|  | L27-R1 | CTCCACCTTGCCGTCGACCAG |  |  |
|  | 16s-F  16s-R | CGCTTTCGTGCCTCAGTGTCAGTGTTGG  GGCGTAAAGCGTGCGTAGGTGGTGGTT | 16S rRNA gene | |
